# Neurofibromatosis type 1-associated plexiform neurofibromas of the neck: topography of lesions and surgical treatment data of 69 patients

**DOI:** 10.1007/s10006-023-01155-5

**Published:** 2023-05-13

**Authors:** Reinhard E. Friedrich, Daniel M. Löhmann

**Affiliations:** Department of Oral and Craniomaxillofacial Surgery, Eppendorf University Hospital, University of Hamburg, Martinistraße 52, 20246 Hamburg, Germany

**Keywords:** Neurofibromatosis type 1, Plexiform neurofibroma, Surgery, Heat map, Neck

## Abstract

**Purpose:**

Plexiform neurofibromas (PNF) are rare tumors arising from peripheral nerve sheath cells. PNF are a hallmark in patients with neurofibromatosis type 1 (NF1), a tumor predisposition syndrome. PNF often grow invasively and destructively, what may complicate surgical treatment. Data on frequency, location, and surgical procedures of patients with NF1-associated FPNF are scarce. This study provides treatment data of NF1 patients.

**Methods:**

Localization and treatment data of 69 NF1 patients with neck PNF were analyzed. Frequency of lesions was recorded in coded colors on schematic neck drawings.

**Results:**

The tumors showed no side preference, were located in the entire area under investigation, and did not respect anatomical units/dermatomes. However, the sternocleidomastoid region was particularly frequently affected. The mean number of surgical measures per patient was 1.33. Complications were extensive swelling, hematoma, and bleeding. Histological assessment usually confirmed the clinical assessment of neoplasm. However, histologic differentiation of PNST reveals differences in between tumors that have been unified in clinical assessment as PNF.

**Conclusion:**

The color-coded, schematic overview of the frequency distribution of surgical neck interventions in NF1 patients with PNF proved a useful tool to gain assessment of preferred treatment needs. The imaging procedure may be suitable for controlling the external aspect of natural tumor development (growth, effects of aging) in the same way as the documentation of the post-surgical course. Treatment plans for patients with these tumors should consider that repeated interventions may be necessary to achieve a longer-term stable result.

## Introduction

The tumor suppressor syndrome neurofibromatosis type 1 (NF1) is a monogenic, autosomal-dominant inherited disease. The penetrability of the syndrome is almost complete. However, the phenotype of NF1 patients is highly variable [1]. The mutation causing NF1 is located on chromosome 17q11.2 [2]. The gene codes for a protein called neurofibromin. Neurofibromin interacts with proteins controlling the rat sarcoma homologue (RAS) in man. In NF1, impaired neurofibromin production is thought to be a major factor in the development of peripheral nerve sheath tumors (PNST) and developmental disorders. Multiple neurofibromas are pathognomonic for NF1 [3, 4]. Schwann cells or their precursors are the origin of tumor cells in NF1-associated PNST [5, 6].

Clinical diagnosis distinguishes mainly two types of neurofibroma: cutaneous and plexiform (PNF). Unlike cutaneous neurofibromas, which are usually numerous and limited in extent to the integument, reaching a maximum size of only a few centimeters in diameter, PNFs are invasive tumors that often destroy the tissues [4, 7, 8]. PNF can cause significant esthetic disfigurement and functional impairment. The tumor can reach significant size and affect development and growth of an entire body region (elephantiasis neuromatosa). PNF is considered a precancerous lesion [1–6]. Data on PNF frequency of the neck region in NF1 is limited because the reports refer to different sources or evaluation criteria of tumors [8]. The differences in the evaluation of tumor diagnosis and management concern both PNST definition and inclusion criteria of the patients, in particular consideration of the genetic background. With reference to other body regions, PNF arising in the head and neck regions is frequently diagnosed in NF1 [3].

Anecdotal reports describe the permanent removal of these tumors with superficial extension and relatively small size. This experience has been evaluated as an argument for early treatment of functionally and esthetically impairing PNF [9]. However, surgical treatment options for PNF are often limited because the tumor already has grown extensively and invasively at the time of treatment decision and a curatively intended, radical-ablative tumor surgery could lead to considerable functional limitations and esthetic impairments.

An orienting classification of the facial tumor spread can be made by assigning the lesions to the cutaneous territories of the respective nerve [10]. However, segmental tumor spread pattern does not imply that the dermatome is completely tumor infiltrated in individual cases, nor does it imply that the tumor will adhere to tumor boundaries because the entity is characterized by slow growth of neoplastic cells.

For over 20 years, the university hospital has had a nationwide outpatient center for the care of neurofibromatosis patients. The annual number of outpatient NF patients treated at the center is around 1100. One of the goals of the center’s activities is to improve diagnostic and therapeutic measures for NF1 patients, with international information exchange [11].

This study determines the extent and surgical treatment procedures of neck PNF in NF1 patients. The aim of the study is to estimate the frequency distribution of tumors assessed as PNF and to provide clinical data for PNST treatment of this manifestation.

## Material and methods

### Patients and procedures

The clinical data of NF1 patients with PNF of the neck were analyzed in a retrospective study**.** All patients were treated by the senior author for neck PNF between 2000 and 2019 at the Department of Oral and Craniomaxillofacial Surgery, Eppendorf University Hospital, Hamburg. All patients met the current diagnostic criteria of NF1 [12]. The surgical measures were primarily based on esthetic and functional reasons in order to reduce the size and shape of disfiguring tumors. Due to the size of the lesions, these were exclusively debulking procedures aimed at restoring the natural contours of the neck by contouring the region of interest. In these cases, esthetic and functional reasons were the predominant indications for surgical intervention. Due to the extent of the tumor, the interventions usually were partial tumor reduction. In some cases, rapid tumor growth was suspicious for malignancy of the mass (malignant peripheral nerve sheath tumor, MPNST) and justified surgical exploration. In the case of nodular tumors, in addition to the characterization of the main lesion, the assessment of the resection margins was the focus of the examination. However, “partial resection” also applies to nodular PNF with macroscopic complete removal, because it is not possible to determine the extent to which the remaining tumor nerve is affected by the nerve sheath tumor in the total length, which is inconspicuous on visual inspection and histological evaluation of border cuts, i.e. showing a benign nerve sheath tumor.

The data was determined from the medical files, operation reports, histological findings, medical reports, and digitally recorded in an anonymous form. The following findings were evaluated: sex, age, number and duration of surgical procedures of the neck, duration inpatient stay, surgery-related complications, histological findings, tumor localization, and number of tumor-affected regions per patient. The duration of the operations was divided into 10-min increments for a simplified overview of data.

### Classifications

**Fig. 1:**
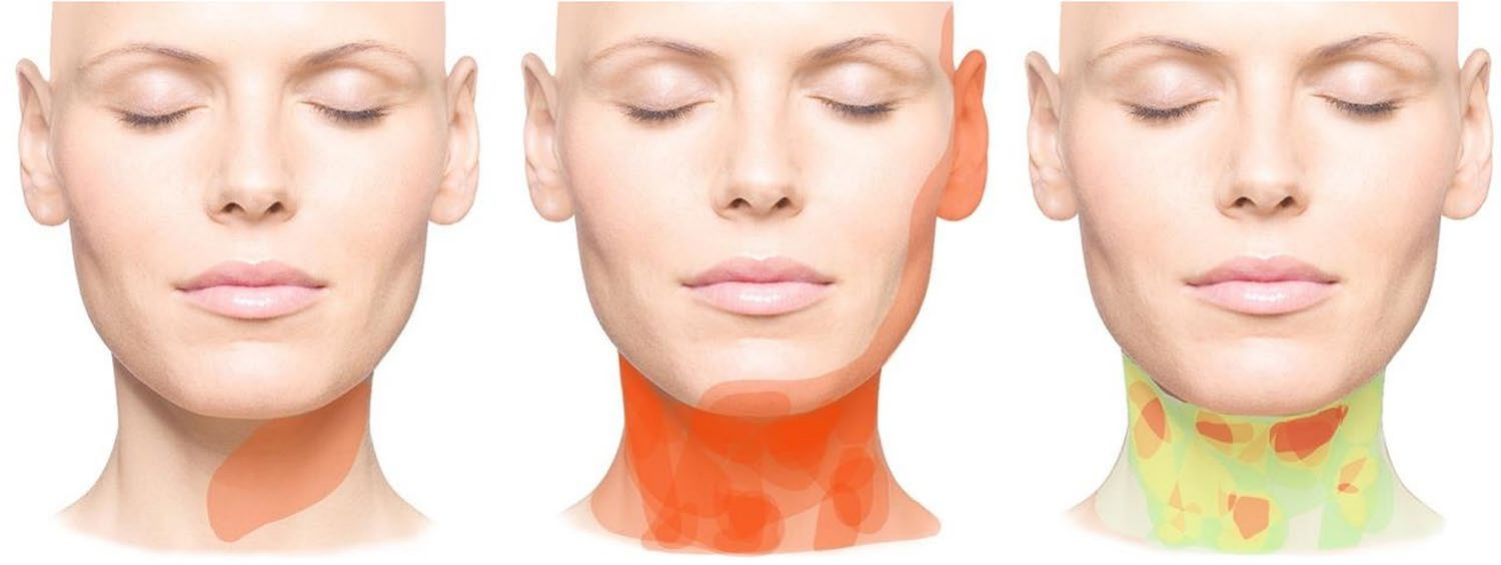
Application of digital tumor extension recording on schematic head drawing. Anterior view of head. Left: Tumor extension of an exemplarily selected case. Middle: Superimposed tumor extensions of the neck in anterior view. Right: Color gradient of tumor distribution frequencies in the same projection (tumor extensions not belonging to the study area were not included in the terminal graphical evaluation)

**Fig. 2:**
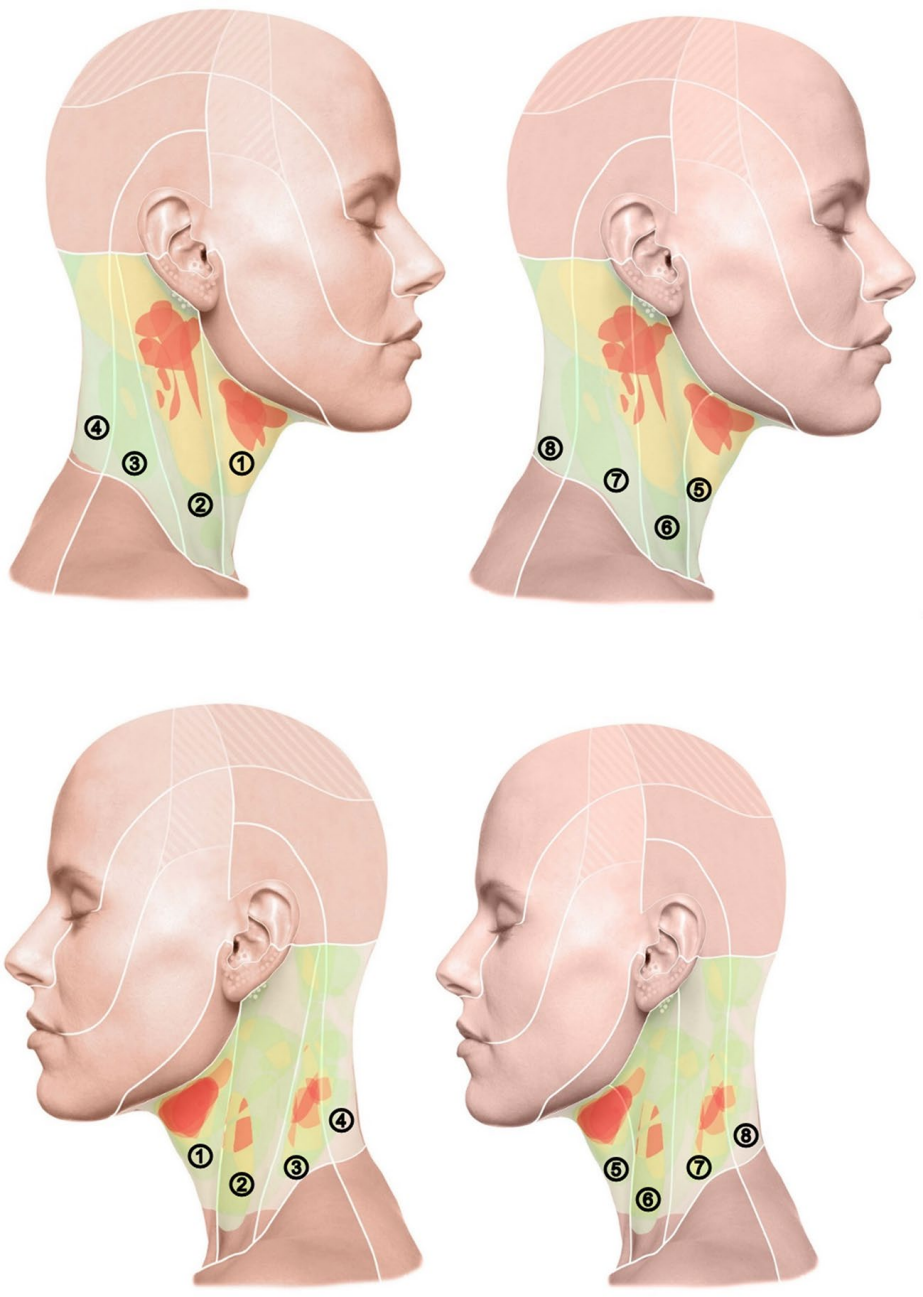
An overview of the frequencies of surgically treated tumor regions of the neck as viewed from the right (top) and left (bottom) on a heat map. The left side of each of the identical figures shows the classification of the surface according to anatomical units; the right side according to dermatomes. The following assignments apply to the numbering of the skin area of the figures: ① Region: cervical anterior, ② Region: sternocleidomastoid, ③ Region: cervical lateral, ④ Region: cervical posterior, ⑤ N. transversus colli, ⑥ N. auricularis magnus, ⑦ N. occipitalis minor, ⑧ Nn. spinales

**Fig. 3:**
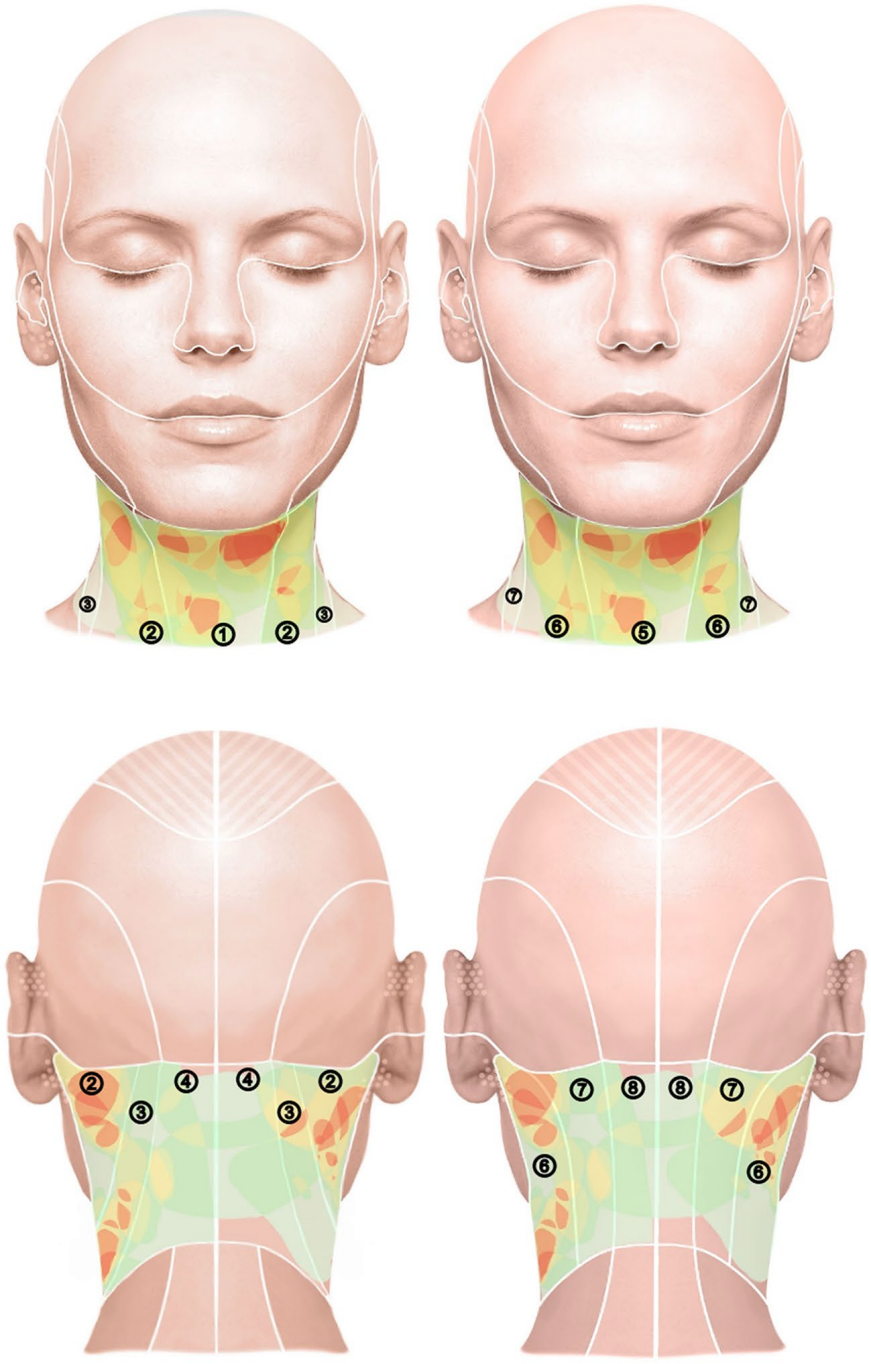
Overview of frequencies of surgically treated tumor regions of the neck as viewed from en face (top) and from the occipital (bottom) on a heat map. The left side of each of the identical figures shows the classification of the surface according to anatomical units; the right side according to dermatomes. The following assignments apply to the numbering of the skin area of the figures: ① Region: cervical anterior, ② Region: sternocleidomastoid, ③ Region: cervical lateral, ④ Region: cervical posterior, ⑤ N. transversus colli, ⑥ N. auricularis magnus, ⑦ N. occipitalis minor, ⑧ Nn. spinales

In *anatomical* classification, the sternocleidomastoid and trapezius muscles divide the neck superficially into four regions (anterior, lateral, and posterior cervical regions and the sternocleidomastoid region), while deeper structures divide it into the so-called cervical triangles (trigonum submandibulare, submentale, caroticum, musculare, and omoclaviculare) [13, 14] (Fig. 1). Inferiorly, the neck is bounded by the clavicle and the superior border of the pars transversa of the trapezius muscle, and superiorly by the inferior border of the mandible, the mastoid process of the temporal bone, and the superior nuchal line of the occipital bone. The anatomical classification of the present evaluation considers the superficial 4 regions. The classification of the neck surface according to *dermatomes* differs from the anatomical classification mainly in the size of the sternocleidomastoid region and its demarcation from the anterior and posterior fields (Figs. 2 and 3). To avoid being influenced by the classifying lines of the skin divisions on the schematic drawings, the lines were inserted into the corresponding files only after the tumors had been drawn onto the digital head and neck surfaces. To include tumors that spread to the skull in the results, a field “adjacent head” was included in the evaluation in addition to the 8 localizations by dermatome/anatomical unit. In addition, photo documentation of the local findings of 39 patients from the archive of the senior author was evaluated. The visible extent of the tumor was manually transferred to a digitalized head and neck scheme [13, 14] (Fig. 1).

The plotted tumor extension was stored in Photoshop Version 2.5 (Adobe; Mountain View, CA, USA). Two sets of images, each modified for and adapted to our own investigations, each with four views (frontal, occipital, lateral right and left) were prepared for each patient. The two sets of figures show lines dividing the head into units or regions [13, 14]. The subdivisions of the skin surface in one set of images are based on the dermatomes of the examination area, the second on the defined anatomical units of the face and head. However, the initial recording of the tumor spread was made using a scheme without scaling in order not to influence the description of the tumor spread by pre-structured diagnostic fields. For each patient with existing photo documentation, the respective extent of the tumor was drawn manually on the scheme. This documentation resulted in different numbers with illustrations of the marked extent of the tumor. The accumulation of the number of tumor localizations was illustrated with a heat map. More commonly affected/treated regions are illustrated in red, less commonly in yellow, and rarely in green. However, the color coding cannot be firmly assigned to a specific number of tumors since different numbers of patients were considered for each side, that is, schematic neck projection. The respective pattern of colors only shows the relationship of tumor frequency for each side and cannot be related to the other sides regarding the number of tumors.

### Complications

Extensive swelling of the surgical site and adjacent regions has occurred in almost all cases and is to be expected in a disease in which tumorous Schwann cells cause the accumulation of interstitial fluid by producing extravasation [15], a process that apparently can be greatly enhanced by mechanical irritation of the surgical intervention. As a result of tumor swelling during debulking procedures, the wound margins often swell, delaying epithelialization of the wound. These findings occur in almost all treatments and are not considered complications. Complications for the purposes of this study are primarily re-bleeding that required revisions, wide wound dehiscence due to unusual tissue swelling and suture insufficiency, and nerve damage.

### Ethics

All procedures performed in this study involving human participants were in accordance with the ethical standards of the institutional and/or national research committee and with the 1964 Declaration of Helsinki and its later amendments or comparable ethical standards. Prior to analysis, data were anonymized, and the investigators studying the radiographs were blinded to diagnosis and individual identity. The investigations of anonymized data were performed in accordance with Hamburgisches Gesundheitsdienstgesetz (Hamburg Health Services Act). This type of investigation does not require the approval of the local ethics committee. The examinations are part of a scientific thesis to fulfil the requirements for the doctorate at the faculty of medicine of the university (DML).

### Statistics

SPSS version 27 (IBM, Armonk, USA) was used to analyze the data. Absolute and relative frequencies, minimum, maximum, median, mean value (MV), and standard deviation (SD) were determined. The data was evaluated by calculating Spearman correlations because no normal distribution of the data could be proven.

## Results

### Age and sex

**Fig. 4:**
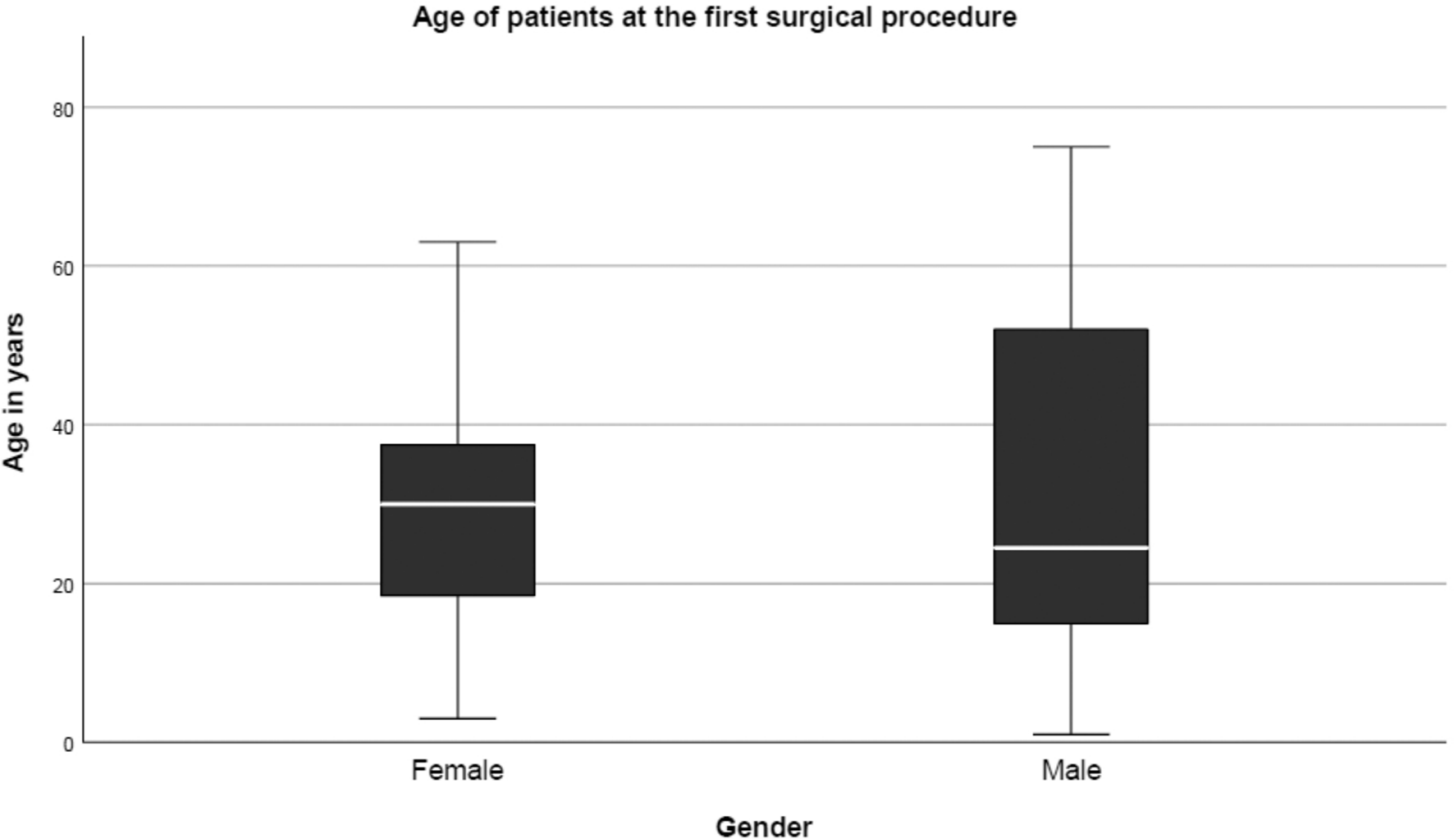
Age of patients with neck plexiform neurofibroma at the time of first surgical procedure

Of the total 69 patients, 26 subjects (37.7%) were male, and 43 subjects (62.3%) were female. At the time of the first surgery, the youngest female patient was 3 years old, and the oldest was 63 years old. Females had a mean age of 29.59 years (SD = 14.38, median 30 years). The youngest male patient was 1 year old at the time of the first surgery, and the oldest was 75 years old. The mean age for males was 30.12 years (SD = 20.87, median 24.5 years). For men, the age at which surgery was performed is distributed over a wider range than for women. For men, the age range above the median is significantly larger than below. In a chronic disease of this localization, the need for surgical treatment in women may be concentrated in a shorter time interval of the life phase (Fig. 4).

### Localization of lesions

Tumors were largely uniformly localized over the study area: Anatomocal regions (bilateral): sternocleidomastoid (32.5%), lateral cervical (25.0%), anterior cervical (25%), and posterior cervical region (17.5%). Anatomical units: left anterior cervical (14.4%), left sternocleidomastoid (13.5%), left lateral cervical (12.5%), right anterior cervical (12.5%), right sternocleidomastoid (11.5%), right lateral cervical (6.7%), and right posterior cervical region (5.8%). Co-involvement of other head regions in tumor infiltration was registered in 13.4%. None of the patients had impaired breathing due to the tumor. Two patients had severe scoliosis and reductions in lung volume resulting in instrumental respiratory support. Extensive tumors with tumorous infiltration and destruction of the musculature resulted in some cases in lowering of the shoulder of the affected side.

### Number of affected regions per patient

The number of regions affected per patient were: 1 (33.3%), 2 (18%), 3 (15.4%), 4 (25.6%), 6 (5.1%), 7 (2.6%).

### Number of surgical procedures per patient

In 69 patients, a total of 92 neck surgeries were performed for PNST. The number of surgeries performed on a patient ranged from one to four (MV = 1.33). Most patients, namely 51 patients (73.9%), were operated only once, while 14 patients (20.3%) were operated twice. The mean value for the difference in patients with multiple surgeries was 739.1 days. The minimum was 13 days, the maximum 4131 days.

## Duration of surgery

**Fig. 5:**
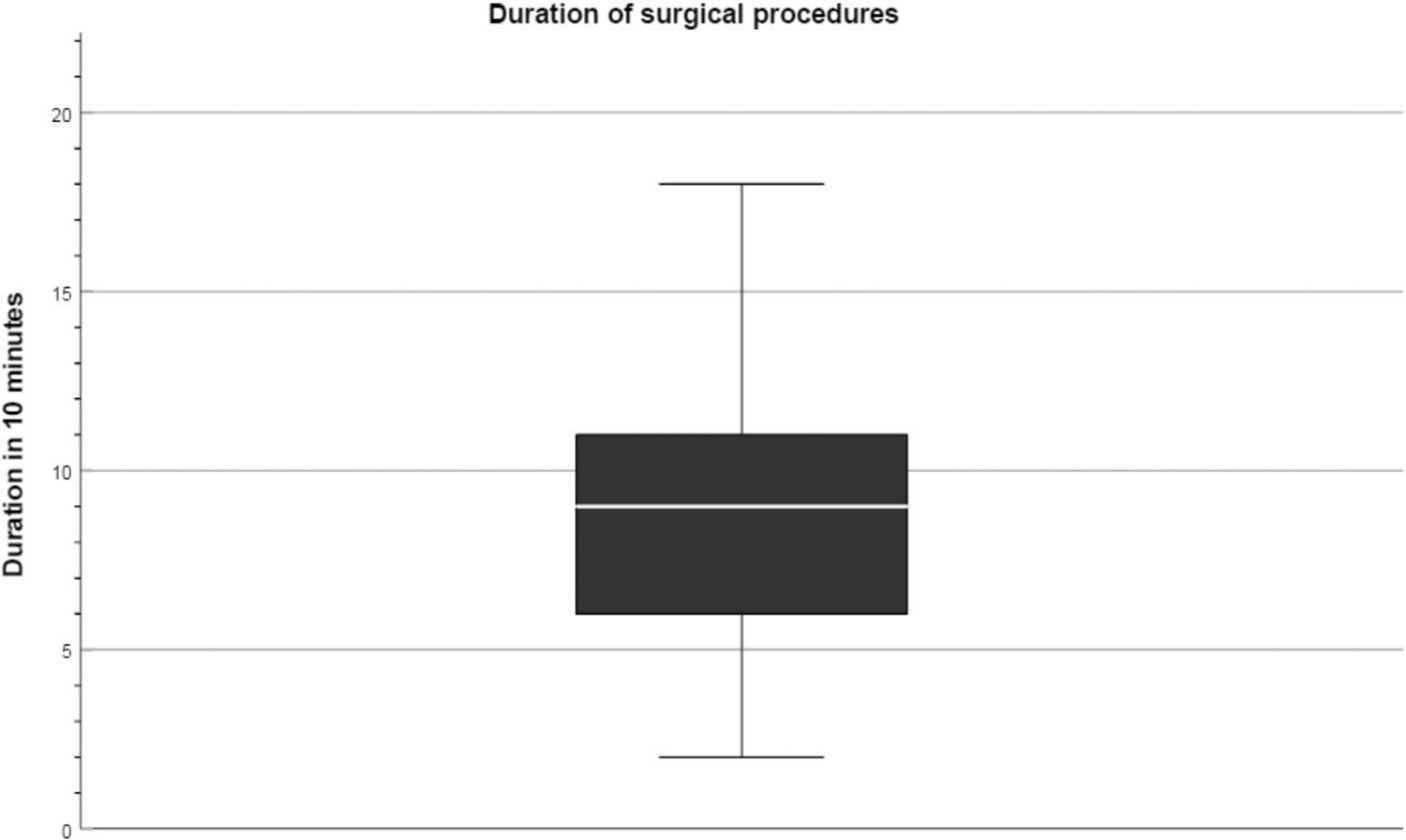
Duration of surgical procedures

The shortest operation duration fell into the second interval, representing a duration between 11 and 20 min. The longest operation lasted 18 increments (171 to 180 min). From the data, the average interval size was 8.7, and the median was 9 (81 to 90 min) (Fig. 5).

### Complications

**Fig. 6:**
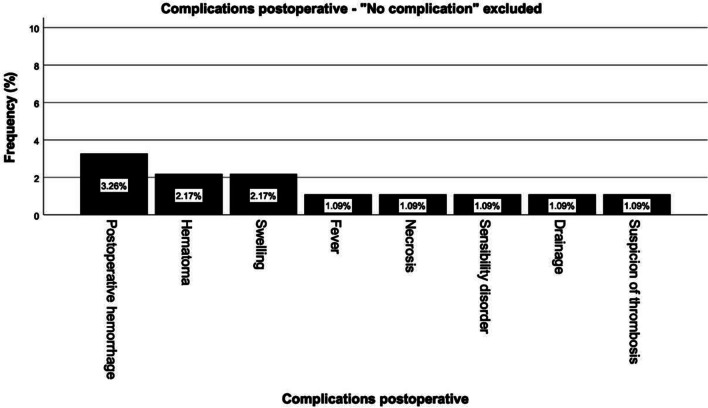
Complications following surgery

In a total of 92 operations, no postoperative complications were recorded in 80 (87%). The most frequent complications were post-operative bleeding in three operations (3.3%), hematoma in two (2.2%), and severe swelling. The complications of fever, necrosis, sensory disturbance, wound secretion, and suspected thrombosis occurred once each (1.1%). In no case, a tracheotomy became necessary. The following diagram does not depict patients without complications to better illustrate relevant findings (Fig. 6).

### Inpatient stay

**Fig. 7:**
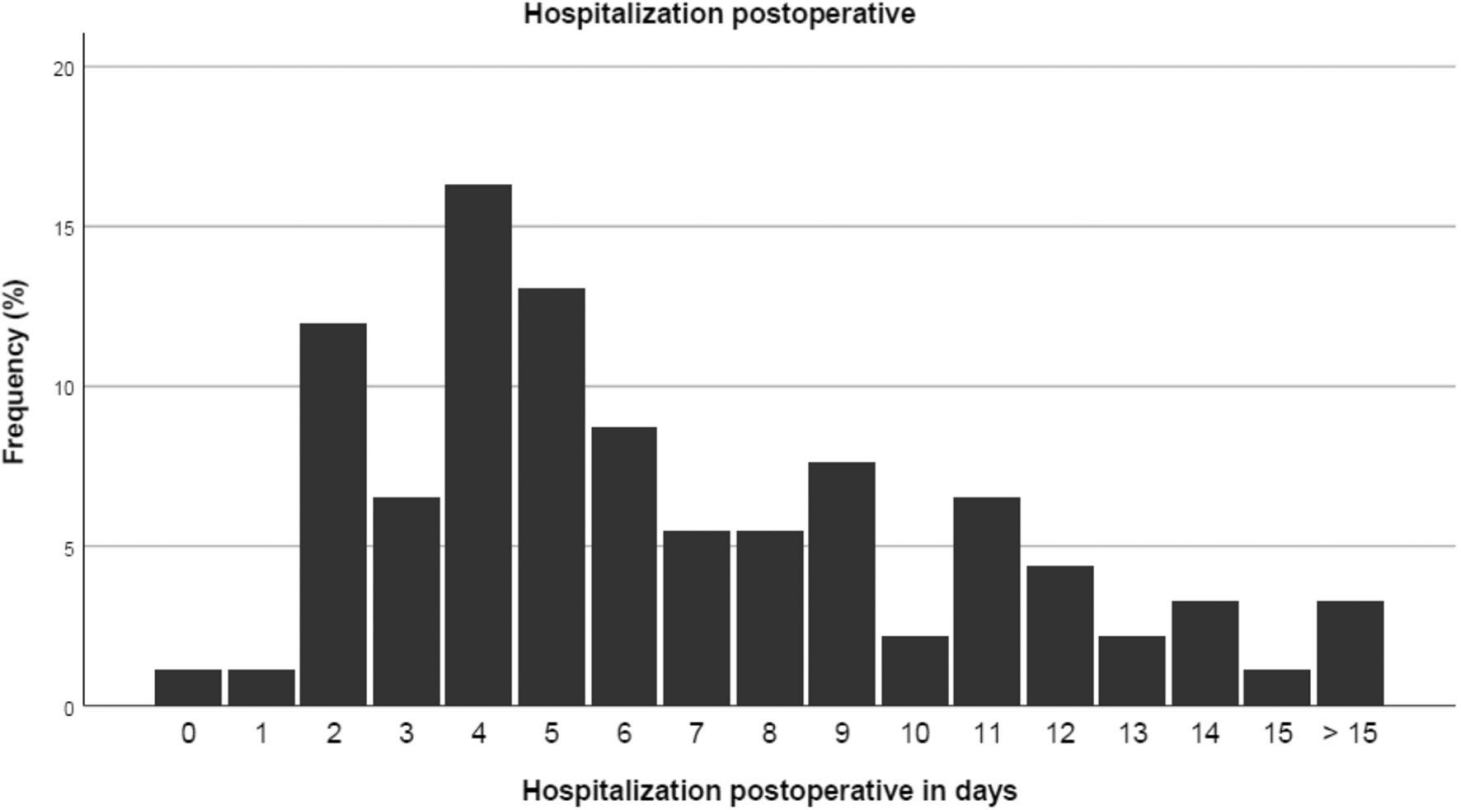
Duration of inpatient stay of NF1 patients

On average, the inpatient stay was 6.91 days (shortest/longest stay: 1/32 day(s)), (Fig. 7).

### Histology

**Fig. 8:**
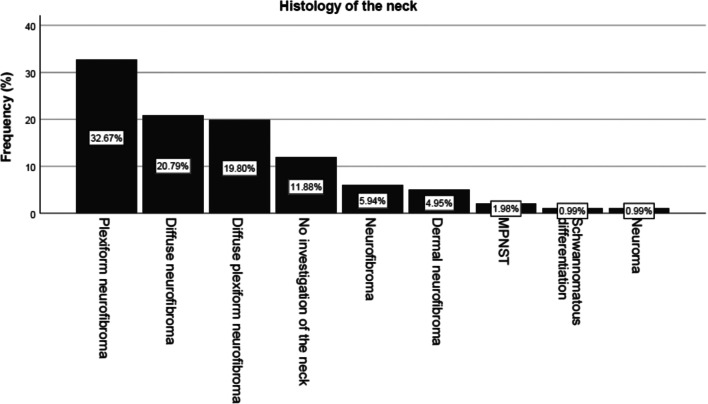
Histology of neck PNST

The histological findings were reevaluated. In the histological findings obtained during the operations, 33 patients (32.7%) were found to have plexiform neurofibromas. The following diagram summarizes the tissue findings: Clearly, there is a difference in the description of tumors in terms of clinical and histologic terminology. The clinical assessment according to tumor extension and consistency (especially the bulky tumors) is often histologically assessed not as a plexiform tumor but as a diffuse neurofibroma. However, histologic evaluation of large specimen relies on selection of representative tissue samples (Fig. 8). Histologic distinctions did not affect the assessment of tumor biology, except for MPNST.

### Imaging of tumor distribution

The photographic documentation of tumor outline of 39 patients constituted the heat map survey of the neck. Because tumors in some cases extend across multiple projections, patients are depicted as having a PNF on more than one body side of the schematic head drawings (front: *n* = 22, left: *n* = 24, right: *n* = 14, back: *n* = 23).

A visual comparison of the two skin surface classifications (anatomical and dermatomes) shows that the hotspots shown fit somewhat better into anatomical divisions, i.e., units. This is clearly visible in several examples: In the view from the right, both the hotspot below the ear and the second hotspot submandibular are divided by a white line according to dermatomes. In contrast, when classified by anatomy, both hotspots are outlined by the white lines. The same is true for the view from the left, in which the submandibular hotspot is divided by dermatomes, which is not the case in the division by anatomy. This phenomenon is also observed in the anterior and posterior views. Based on this, the manifestation of plexiform neurofibromas was analyzed in more detail for the individual surfaces of the anatomical units. Thus, it can be assumed that defining the anatomical neck PNF unit is relevant for surgical assessment of tumor spread. However, the tumors also cross the boundaries of the units in the anatomical classification of the skin surface (Figs. 2 and 3).

### Correlations

In a correlation analysis, the parameters of number of operations, number of affected regions, and length of inpatient stay after surgery were compared. None of the correlated parameters showed meaningful significance (Number of regions and number of surgical procedures: correlation coefficient (CC) = 0.032, *p* = 0.816; number of regions and inpatient stay: CC = 0.143, *p* = 0.302; number of surgical procedures and inpatient stay: CC = −0.106, *p* = 0.447).

## Discussion

The study analyzes the surgical treatment data of NF1 patients with PNF of the neck. Tumors in this location can be managed within a routine program of surgical clinics.

### Treatment data

No sex-specific clustering of tumor localizations was observed. Apparently, a few interventions are sufficient to achieve an improvement in appearance and function. Overall, the proportion of malignant tumors is low, which agrees with the distribution pattern of MPNST. The accumulation of surgical interventions to a certain age range corresponds to the known experience that tumor growth takes place particularly in youth and early adulthood and that the need for treatment already exists in this period of time. The boxplots indicate that the main phase of surgical treatment is preferably around 20–40-year-old patients. The correlation analysis shows no relationship between tumor extent (number of regions), number of surgical measures, and the duration of inpatient stay. Significant deviations from this estimate are to be expected when very large tumors that have grown far beyond anatomic units are treated surgically; when interventions are planned that are combined with skeletal/intraspinal measures of the region; and in cases with malignant peripheral nerve sheath tumors arising from neck PNF [16]. There are obvious differences in the tumor types to be treated in the respective specialist clinics. Thus, plastic surgical interventions are more common in some departments than in other departments that focus on the exploration of tumors of the brachial plexus. It is currently unclear whether the manifestations of the lesions differ depending on the genetic background of the patient. However, non-syndromic neoplasms were more common than NF1-associated ones in PNST of the brachial plexus [16]. Inpatient treatment time and complication pattern reflect follow-up characteristics of a disease with increased postoperative bleeding tendency and delayed wound healing [17, 18]. The length of the inpatient stay is explained by the delayed wound healing, the risk of secondary bleeding from the wounds, and the fact that outpatient specialist care for the patient at home is often not guaranteed. The complication rate is about 13% and justifies inpatient care of patients as well as individually adapted length of hospital stay. Bleeding from the PNF can influence the extent and duration of surgical measures [18, 19]. Wound healing results are esthetically satisfactory in many cases [20].

### Heat map-aided illustration of preferred sides of surgically treated tumor regions

A graphical representation of tumor localization clustering illustrates approximately bilaterally symmetrical distribution of the tumors. Within the affected side of the body, patterns of tumor spread are recognizable, which were best described in this study by assignment to anatomical units. The examination confirms that the tumors have a segmental spread [10]. Furthermore, the preferential localization of the tumors in the sternocleidomastoid region is evident from the heat map. The clustering corresponds to data in the literature in which the surgical treatment results of brachial plexus PNF have been analyzed [15, 21, 22]. The description of cutaneous PNF distribution of the neck corresponds to results for the PNF distribution pattern developing in the extremities [23, 24]. In the regions of the trunk and extremities, segmental distribution of tumors across dermatomes and units was obvious [23–25]. In contrast, description of tumor distribution according to dermatomes of the trigeminal nerve has proven useful for topographical classification of facial PNF [26, 27]. Whether the classification according to anatomical units for PNF of the neck is suitable for the analysis and treatment planning of NF1 patients in general should be tested on a larger study group. In addition to analyzing the frequency of tumor manifestations, the examination procedure is suitable for assessing the individual course of the disease as long as the tumors cause externally visible changes of the neck (e.g., to objectify tumor growth).

### Defining PNF and impact of PNST diagnosis on estimating tumor progression and treatment options

Histologic evaluation of the resected specimens confirmed the diagnosis of PNST in most cases [16, 17]. However, there are differences in the use of the term “plexiform” based on medical specialties [28–34]. The histological distinction is based on the relationship between the axon and the tumorous Schwann cells. In histological definition, the diagnosis “plexiform” has no relationship to tumor size [24]. In contrast, in the clinical-diagnostic field, the term “plexiform” is predominantly used to describe both large tumors with infiltrative growth patterns (lumpy masses) and nodular PNSTs arising, for example, in the plexus (nodular PNF). The differences between the histologic definition of PNST and clinical practice indicate the current state of the diagnostic art, in which the term “PNF” is not clearly defined [8]. Among the variant PNSTs are especially sack-like tumors, often infiltrating only the subcutis, which are regularly classified histologically as diffuse neurofibroma/diffuse plexiform neurofibroma. Clinical assessment often leads to the designation of superficial neurofibroma [30, 33]. In most, but not all cases, this type of spread is considered a benign tumor [34]. The problem of selecting representative tissue samples for general diagnosis of a large tumor specimen has already been pointed out [23].

A recently published study mapped the distribution of plexiform neurofibroma on the body surface [25]. The number of head and neck PNF was high (19.2%). The PNSTs were addressed as diffuse plexiform neurofibromas. However, the diagnosis of PNF was not further specified by a histological or clinical definition of the entity. In addition, the tumor distribution was not classified according to dermatomes or anatomic units. Earlier studies suggest that head and neck PNF are very common tumor sites in NF1 patients requiring surgical intervention [3, 16, 17, 35, 36].

The imaging procedure was used to specify tumor localization and treatment needs of a group of patients suffering from specific tumors preferentially arising in NF1, a tumor predisposition syndrome. However, the selection criterion, i.e., analyzing surgically treated patients, must not lead to generalization of tumor distribution and its frequency in the region studied. However, the study may help to more precisely specify regions that are particularly stressful for patients and prompt them to seek surgical assistance and to support therapy planning.

The differences between histological evaluation of the tumor and its clinical assessment were revealed in this study (Fig. 8). Large, dewlap-like tumors that have been clinically assessed as PNFs on the basis of extension and disfigurement can be diagnosed as “diffuse” or “plexiform/diffuse” as well as “plexiform” neurofibromas on tissue analysis. These tumors frequently appear like superficial (often sagging) tumors due to the obvious deformation of the body region or shape. The radiologically defined distinguishing feature of superficial neurofibromas is that these do not invade the deeper layers of the body [29] and therefore constitute a separate entity [30]. However, the conclusion derived from this classification cannot be generalized. This type of tumor can invade the muscles of the neck. It is possible that this progressive phenotype of the superficial PNF is age-dependent.

## Conclusion

PNF of the neck region in NF1 patients is largely equally distributed across anatomic regions. Surgical treatment of plexiform neurofibroma in the region should be planned considering the extent of the individual tumor and the associated morbidity. Centers with specifications for the care of NF1 patients can offer interdisciplinary therapeutic concepts. Frequency analysis of surgical procedures using a color-coded overlay (heat map) of surgically treated tumor regions detects preferentially affected regions in need for surgical therapy. The superimposition technique of photographic findings is suitable for surgical follow-up of PNF treatment but also for the assessment of contour changes of the affected region in the natural course of the disease.

## Data Availability

The datasets generated during and/or analyzed during the current study are available from the corresponding author on reasonable request.

## References

[1] Ferner RE, Gutmann DH (2013). Neurofibromatosis type 1 (NF1): diagnosis and management. Handb Clin Neurol.

[2] Kluwe L, Friedrich RE, Korf B, Fahsold R, Mautner VF (2002). NF1 mutations in neurofibromatosis 1 patients with plexiform neurofibromas. Hum Mutat.

[3] Huson SM, Compston DA, Harper PS (1989). A genetic study of von Recklinghausen neurofibromatosis in south east Wales. II. Guidelines for genetic counselling. J Med Genet.

[4] Friedman JM, Birch PH (1997). Type 1 neurofibromatosis: a descriptive analysis of the disorder in 1,728 patients. Am J Med Genet.

[5] Kluwe L, Friedrich R, Mautner VF (1999). Loss of NF1 allele in Schwann cells but not in fibroblasts derived from an NF1-associated neurofibroma. Genes Chromosom Cancer.

[6] Kluwe L, Friedrich RE, Mautner VF (1999). Allelic loss of the NF1 gene in NF1-associated plexiform neurofibromas. Cancer Genet Cytogenet.

[7] Collins-Sawaragi YC, Ferner R, Vassallo G, De Agrò G, Eccles S, Cadwgan J, Hargrave D, Hupton E, Eelloo J, Lunt L, Tang V, Burkitt Wright E, Lascelles K (2022). Location, symptoms, and management of plexiform neurofibromas in 127 children with neurofibromatosis 1, attending the National Complex Neurofibromatosis 1 service, 2018-2019. Am J Med Genet A.

[8] Fisher MJ, Blakeley JO, Weiss BD, Dombi E, Ahlawat S, Akshintala S, Belzberg AJ, Bornhorst M, Bredella MA, Cai W, Ferner RE, Gross AM, Harris GJ, Listernick R, Ly I, Martin S, Mautner VF, Salamon JM, Salerno KE, Spinner RJ, Staedtke V, Ullrich NJ, Upadhyaya M, Wolters PL, Yohay K, Widemann BC (2022). Management of neurofibromatosis type 1-associated plexiform neurofibromas. Neuro Oncol.

[9] Friedrich RE, Schmelzle R, Hartmann M, Fünsterer C, Mautner VF (2005). Resection of small plexiform neurofibromas in neurofibromatosis type 1 children. World J Surg Oncol.

[10] Lubinsky MS (2006). Non-random associations and vascular fields in neurofibromatosis 1: a pathogenetic hypothesis. Am J Med Genet A.

[11] Carton C, Evans DG, Blanco I, Friedrich RE, Ferner RE, Farschtschi S, Salvador H, Azizi AA, Mautner V, Röhl C, Peltonen S, Stivaros S, Legius E, Oostenbrink R, ERN GENTURIS NF1 Tumour Management Guideline Group (2023). ERN GENTURIS tumour surveillance guidelines for individuals with neurofibromatosis type 1. EClinicalMedicine.

[12] Legius E, Messiaen L, Wolkenstein P, Pancza P, Avery RA, Berman Y, Blakeley J, Babovic-Vuksanovic D, Cunha KS, Ferner R, Fisher MJ, Friedman JM, Gutmann DH, Kehrer-Sawatzki H, Korf BR, Mautner VF, Peltonen S, Rauen KA, Riccardi V, Schorry E, Stemmer-Rachamimov A, Stevenson DA, Tadini G, Ullrich NJ, Viskochil D, Wimmer K, Yohay K, Huson SM, Evans DG, Plotkin SR, International Consensus Group on Neurofibromatosis Diagnostic Criteria (I-NF-DC) (2021). Revised diagnostic criteria for neurofibromatosis type 1 and Legius syndrome: an international consensus recommendation. Genet Med.

[13] Radlanski RJ, Wesker KH (2012). Das Gesicht. Bildatlas klinische Anatomie.

[14] Aumüller G, Bob A, Bob B (2007). Duale Reihe Anatomie. Duale Reihe Anatomie.

[15] Hagel C, Behrens T, Prehm P, Schnabel C, Glatzel M, Friedrich RE (2012). Hyaluronan in intra-operative edema of NF1-associated neurofibromas. Neuropathology.

[16] Donner TR, Voorhies RM, Kline DG (1994). Neural sheath tumors of major nerves. J Neurosurg.

[17] Janes LE, Sabino J, Matthews JA, Papadimitriou JC, Strome SE, Singh DP (2013). Surgical management of craniofacial neurofibromatosis type 1 associated tumors. J Craniofac Surg.

[18] Wolkenstein P, Mitrofanoff M, Lantieri L, Zeller J, Wechsler J, Boui M, Revuz J, Mansat E, Stalder JF (2001). Bleeding: a complication of neurofibromatosis 1 tumors. Arch Dermatol.

[19] Hivelin M, Plaud B, Hemery F, Boulat C, Ortonne N, Valleyrie-Allanore L, Wolkenstein P, Lantieri L (2016). Low rates of blood transfusion in elective resections of neurofibromas in a cohort study: neurofibroma length as a predictor of transfusion requirement. Plast Reconstr Surg.

[20] Miyawaki T, Billings B, Har-Shai Y, Agbenorku P, Kokuba E, Moreira-Gonzalez A, Tsukuno M, Kurihara K, Jackson IT (2007). Multicenter study of wound healing in neurofibromatosis and neurofibroma. J Craniofac Surg.

[21] Kim DH, Murovic JA, Tiel RL, Moes G, Kline DG (2005). A series of 397 peripheral neural sheath tumors: 30-year experience at Louisiana State University Health Sciences Center. J Neurosurg.

[22] Desai KI (2017). The surgical management of symptomatic benign peripheral nerve sheath tumors of the neck and extremities: an experience of 442 cases. Neurosurgery.

[23] Friedrich RE, Diekmeier C (2017) Peripheral nerve sheath tumors of the upper extremity and hand in patients with neurofibromatosis type 1: topography of tumors and evaluation of surgical treatment in 62 patients. GMS Interdiscip Plast Reconstr Surg DGPW 6. 10.3205/iprs000117 10.3205/iprs000117 PMC5717919 29214122

[24] Friedrich RE, Tuzcu CT (2021). Surgery for peripheral nerve sheath tumours of the buttocks, legs and feet in 90 patients with neurofibromatosis type 1. In Vivo.

[25] Ehara Y, Koga M, Imafuku S, Yamamoto O, Yoshida Y (2020). Distribution of diffuse plexiform neurofibroma on the body surface in patients with neurofibromatosis 1. J Dermatol.

[26] Friedrich RE, Lehmann JM, Rother J, Christ G, Zu Eulenburg C, Scheuer HT, Scheuer HA (2017). A lateral cephalometry study of patients with neurofibromatosis type 1. J Craniomaxillofac Surg.

[27] Greig AV, Kirkpatrick NA, Joshi N, Kelly M, Waterhouse N (2009). Giant hemifacial plexiform neurofibroma arising from trigeminal ganglion. J Craniofac Surg.

[28] Friedrich RE, Korf B, Fünsterer C, Mautner VF (2003). Growth type of plexiform neurofibromas in NF1 determined on magnetic resonance images. Anticancer Res.

[29] Mautner VF, Hartmann M, Kluwe L, Friedrich RE, Fünsterer C (2006). MRI growth patterns of plexiform neurofibromas in patients with neurofibromatosis type 1. Neuroradiology.

[30] Lim R, Jaramillo D, Poussaint TY, Chang Y, Korf B (2005). Superficial neurofibroma: a lesion with unique MRI characteristics in patients with neurofibromatosis type 1. AJR Am J Roentgenol.

[31] Scheithauer BW, Woodruff JM, Erlandson RA (1999). Tumors of the peripheral nervous system. Third series, fascicle 24 (AFIP atlas of tumor pathology).

[32] Friedrich RE, Nörnberg LKN, Hagel C (2022). Peripheral nerve sheath tumors in patients with neurofibromatosis type 1: Morphological and immunohistochemical study. Anticancer Res.

[33] O’Keefe P, Reid J, Morrison S, Vidimos A, DiFiore J (2005). Unexpected diagnosis of superficial neurofibroma in a lesion with imaging features of a vascular malformation. Pediatr Radiol.

[34] Inoue T, Kuwashiro M, Misago N, Narisawa Y (2004). Superficial malignant peripheral nerve sheath tumor arising from diffuse neurofibroma in a neurofibromatosis type 1 patient. J Dermatol.

[35] Hsu CK, Denadai R, Chang CS, Yao CF, Chen YA, Chou PY, Lo LJ, Chen YR (2022). The number of surgical interventions and specialists involved in the management of patients with neurofibromatosis type I: a 25-year analysis. J Pers Med.

[36] Neville HL, Seymour-Dempsey K, Slopis J, Gill BS, Moore BD, Lally KP, Andrassy RJ (2001). The role of surgery in children with neurofibromatosis. J Pediatr Surg.

